# Association amid the comorbidity of Diabetes Mellitus in patients of Active Tuberculosis in Pakistan: A matched case control study

**DOI:** 10.12669/pjms.37.3.3274

**Published:** 2021

**Authors:** Nadia Khalid, Farah Ahmad, Farhan Muhammad Qureshi

**Affiliations:** 1 Dr. Nadia Khalid, MBBS. Senior Lecturer, Department of Community Health Sciences, Bahria University Medical & Dental College, Karachi, Pakistan; 2 Dr. Farah Ahmad, MCPS-HCSM, MSBE. Associate Director, Health Care System Management, College of Physicians & Surgeons, Karachi, Pakistan; 3 Dr. Farhan Muhammad Qureshi, MS - Public Health & Health Promotion Assistant Professor, Department of Community Health Sciences, Karachi Institute of Medical Sciences, Malir Cantt, Karachi, Pakistan

**Keywords:** Association, Co-morbidity, Diabetes mellitus, Tuberculosis, Tuberculous infection

## Abstract

**Objectives:**

This study aims to test the association between diabetes and tuberculosis.

**Methods:**

It is a matched case control study conducted in tertiary care hospitals in 2019-2020. Cases and controls were 144 each, selected on the basis of an odds ratio of 2 at 95% confidence interval with a significance level of 5%. Cases of pulmonary tuberculosis were selected through consecutive sampling technique, either visiting OPD or admitted in hospital. Controls were taken from the general population and frequency matching was done based on age, gender and socioeconomic status. Data was collected through structured questionnaire after taking written consent. Data was analyzed on SPSS version 23. Binary Logistic regression model was applied for finding association between the risk factors and the disease. P value <0.05 was considered statistically significant.

**Results:**

Out of all cases and controls, 45% and 20% were diabetics respectively. The association between the risk factors and tuberculosis was estimated by univariate analysis, positive association was found between diabetes and tuberculosis (OR= 3.32), a high frequency of diabetes in cases as compared to controls were observed with a highly significant p- value (<.001).

**Conclusions:**

This study provides evidence for a strong positive association between tuberculosis and diabetes.

## INTRODUCTION

Tuberculosis (TB) ranked as one of the leading causes of death, with an estimated 10.4 million cases across the globe in 2015.^1^ Among 22 countries with generalized TB epidemics, Pakistan ranked 5^th^ due to its growing epidemics.^2^ Risk become high in vulnerable individuals and population having diseases which lowers the immunity such as AIDS, cancer, malnutrition and Diabetes Mellitus (DM) etc. DM is considered to be a well-known risk factor for Tuberculosis. However, both DM and TB are an immense public health problem worldwide, individually and as co-morbid. In developing part of the world where TB is endemic, prevalence of DM is rising that shows the link between TB and DM.^3^ The risk of TB becomes three folds in individuals having DM and an increase in overall, global incidence of tuberculosis. Further, despite controlled incidence of tuberculosis in developed countries the association between TB and DM has been proven.^3^ However, its association is quite complex and it is still unclear that DM increases the vulnerability to develop TB infection that cause the development of active disease. Although, there is a lacking of strong supportive evidence that explain the actual mechanism of action of this association, compromised cell-mediated immunity or neutrophil function are considered to play a major role in its association.^3^

DM and TB are more prevalent in low-income countries due to unawareness, weak health infrastructure, poverty and poor living conditions, as occurrence of anyone causes individual immunocompromised and predispose to other.^4^ Pulmonary tuberculosis is the most common type that is associated with Type-II Diabetes as compared to Type-I diabetes.^5^ Evidence shows that individuals having DM is at high risk of active tuberculosis with an associated poor compliance with antituberculosis therapy (ATT) as well as subsequent risk of recurrence of TB.^6^ Also, both TB and DM have adversely impact on each other if occurred simultaneously with resultant bad treatment response to antituberculosis therapy and uncontrolled blood sugar levels. Hence, the association of diabetes and tuberculosis emerges as growing challenge of global concern in context of TB control measures.^7^

In Mediterranean and American region of WHO, the prevalence rate is 17% followed by Europe (15%) and South East Asian region (14%).^5^,^8^ Moreover, countries where association found high between these two comorbidities are Bangladesh, China, India and Pakistan.^8^ Numerous behavioral and sociodemographic risk factors are associated with the comorbidity of diabetes and tuberculosis, especially low socioeconomic status, tobacco and alcohol consumption in developing countries. However, both genders are at equal risk of developing both diseases.^9^ The double burden of both diseases especially in lower income countries, facing the increasing prevalence of DM along with tuberculosis, now an area of growing challenge in the field of public health and; specialists have concerns about merging epidemic of both diseases. Thus, the current study was designed to find out the association of diabetes in patients with tuberculosis.

## METHODS

This was a matched case control study conducted in two tertiary care hospitals of Karachi city from November 2019 – May 2020. Sample size calculated was; n=144 for cases and controls each, on the basis of an odds ratio of 2 at 95% confidence interval with a significance level of 5% and considering proportion of Diabetes mellitus in controls as 11% in Pakistan for 2017. The proportion of cases to controls was 1:1.

Cases of pulmonary tuberculosis were selected through consecutive sampling technique, the criteria for case selection were patients (age ≥18 years) presenting in the OPD and later admitted in the hospitals, with new active or previously diagnosed tuberculosis that was based on (sputum smear microscopy, chest X-ray, clinical evidence of TB and decision by the clinician to treat with a full-course of anti-TB therapy. Controls were taken from the general population and frequency matching was done based on age, gender and socioeconomic status. Diabetes, the risk factor in both cases and controls was diagnosed by checking blood sugar levels according to the WHO classification.^10^

Patients were interviewed after explaining the purpose of study when they came to seek the medical care in OPD and then admitted in ward. Structured questionnaire was developed through extensive literature search and evidence-based risk factors for tuberculosis were added comprising of age, gender, education status, malnutrition, smoking and socio-economic factors. Data was analyzed in SPSS version 23. Descriptive analysis was in the form of mean±standard deviation for numeric variables and frequencies and percentages for categorical variables. Univariate analysis was done through Odds ratio calculation expressed in p value and confidence interval for all the risk factors and disease status. Bivariate analysis was conducted between outcome and only risk factors which came out to be significant in Univariate analysis through binary logistic regression model. P value <0.05 was considered statistically significant.

### Ethical Consideration:

Ethical consideration (ERC53/2019) was taken from the Ethical Review Committee (ERC) of Bahria University Medical & Dental College (BUMDC), Karachi. Data collection process was started after receiving the ERC letter from the institution. Patients were verbally explained the motive of the research before signing the informed consent. Anonymity was maintained with all information kept confidential.

## RESULTS

The final sample size comprises of n= 97 cases of tuberculosis and n=150 controls. The controls were matched through frequency matching conducted on the basis age, gender, education and socioeconomic status (Table-I). The mean age of cases was 43.7±13.5 and 41.9±11.2 among controls. Out of all cases and controls, 45% and 20% were diabetics respectively. In cases, 42% were males with diabetes and 48% were females.

**Table I:** Sociodemographic Variables in Cases and Controls.

Variable		Cases N (%)	Controls N (%)
Gender	Female	47(48.5)	76 (48.7)
	Male	50 (51.5)	74(47.3)
Income	<30.000	83(85.5)	100 (66.6)
	>30,000	14(14.4)	50 (33.3)
Education	Illiterate	35 (36.1)	35 (23.3)
	Primary	15 (15.5)	34 (22.7)
	Matric	36 (37.1)	47 (31.3)
	Graduate	11 (11.3)	34 (22.7)

When gender was compared, males were n=50(51.5%) in cases and in controls n=74(47.3%) whereas females were almost in same proportion as in cases n=47(48.5%) and in controls n=76(48.7%). When educational status of participants was assessed, majority of patients were matriculate n=36(37.1%). Same findings were observed among controls where majority were matriculate n= 47 (31.3%). When Diabetes was assessed in both cases and controls, n=44(45%) was diabetic in cases and n=30(20%) in controls.

Univariate analysis was done to estimate the association between risk factors and tuberculosis. Overall, P value was observed highly significant (<.001), showing positive association between diabetes mellitus and tuberculosis (OR=3.32). Also, family history showed a positive association with an odds ratio of 2.7 and a significant p-value (0.0008) as a risk factor in 38% and 18% among cases and controls respectively. Moreover, the odds ratio for malnutrition was calculated as 2.1 with a p-value of 0.003 showing significant statistical association. Regarding the association with socioeconomic status, income was found less than 30,000 in cases n= 83(85%) and in controls n=100(66%) with a significant p-value (.003) and odds ratio of 2.9.

**Table II:** Univariate analysis of diabetes and tuberculosis.

S #	Variables		Cases	Controls	Odds Ratio	CI	P-Value
1.	Diabetes	Yes	44	30	3.32	1.88--5.8	0.0001
		No	53	120			
2.	Family History	Yes	37	28	2.7	1.5—4.8	0.0008
		No	60	122			
3.	Malnutrition	Yes	12	1	2.1	2.67—1.65	0.003
		No	85	149			
4.	Smoking	Yes	30	22	1.3	0.7—2.6	0.37
		No	67	66			
5.	Air pollution	Yes	17	28	0.94	0.48—1.84	0.8
		No	78	122			
6.	Income	<30,000	83	100	2.9	1.53—5.7	0.003
		>30,000	14	50			

**Table III:** Binary logistic regression analysis of cases and controls.

	B	S.E.	Wald	df	Sig.	Exp(B)	95% C.I. for EXP(B)	

							Lower	Upper
Family History (1)	0.685	0.328	4.376	1	0.036	1.985	1.044	3.772
Risk of Malnutrition (1)	2.641	1.066	6.137	1	0.013	14.033	1.736	113.432
Diabetes (1)	1.030	0.308	11.156	1	0.001	2.8	1.53	5.12
Income (1)	0.932	0.356	6.851	1	0.009	2.539	1.264	5.103
Constant	-1.772	0.340	27.175	1	0.000	0.170		

a. Variable(s) entered on step 1: Family history, Risk of malnutrition, Diabetes, Income.

The logistic regression was performed to test influence of family history, risk of malnutrition, diabetes and income on tuberculosis. Results indicated that the predictor model provided a statistically significant improvement over the constant-only-model, χ2 (4, *N*= 247) = 45.4, *p* = 0.000. The Nagelkerke R2 indicated that the model accounted for 23% of the total variance. The correct prediction rate was about 70%. The Wald tests showed that family history, risk of malnutrition, diabetes and income all significantly predicted Tuberculosis.

## DISCUSSION

In this research on the DM-TB association, results revealed that considerable proportion of patients with tuberculosis having comorbidity with DM. Diagnosed and uncontrolled cases of DM are more prone to develop active tuberculosis in comparison of non-diabetics.^11^ The current study identified strong positive DM-TB association as co-morbid due to increased frequency of diabetes in patients of tuberculosis as compared to controls. Nations, belong to the developing part of the world is continuously struggling to combat these chronic conditions due to high prevalence and growing epidemics because of rapid economic, social and life style changes.^12^ Studies conducted in Brazil, China and Peru are in line with the results of this research and observed a positive DM-TB association with much higher results.^7^,^13^-^15^ Anti-tuberculosis medicines worsen the glycemic control and diabetes can cause relapse or worsen the clinical course of TB, that might be a reason of strong positive association, as both conditions are more prevalent in developing countries individually or as comorbid.^13^,^14^ These findings are further strengthened by research conducted to find out the DM-TB association and have reported similar findings especially in high endemic areas of tuberculosis where diabetes triples the risk of disease.^16^

Further, certain sociodemographic and behavioral covariates of the participants such as monthly income, family history, nutritional status, smoking and indoor living environment are found to be associated with DM-TB comorbidity. The results from the present study are consistent with a case control study conducted on risk factors associated with TB.^17^ Although, smoking is considered to be a potent risk factor for tuberculosis and a major public health issue in Pakistan, but unexpectedly, the current study wasn’t found a statistical association within the comorbidity of tuberculosis and diabetes with the p-value of 0.37. On the contrary, study conducted in Romania revealed that smokers are two times more likely to develop tuberculosis as compared to non-smokers.^18^ However, the unpredictable result of this research might be due to false information provided by the patients regarding smoking habits.

Even though the tuberculosis is not endemic in developed countries, diabetes can cause reactivation of the latent cases of tuberculosis as well as hamper the control measures.^19^ Systematic reviews and meta-analysis also showed strong association between diabetes and tuberculosis and declared two to three times increase risk of tuberculosis among those who have diabetes as compared to non-diabetics^20^ that supports the outcome of the current research. This study exhibited the supporting evidence of substantial risk of developing tuberculosis among diabetic patients as compared to non-diabetics. Hence, the increasing prevalence of DM leads to be a potential threat against the control measures of tuberculosis especially in middle- and low-income countries.

### Limitations of the study:

Lack of resources and time pressure due to short study duration were the major factors that caused hindrance to achieve the estimated calculated sample size.

## CONCLUSION

This study provides evidence for a strong positive association between TB and DM that point to another challenge for the control measures of tuberculosis. To avoid threatening of the dual disease burden, the strategic planning must be considered with integrated actions in order to prevent the consequences. Further, research on a large scale is recommended to evaluate and analyze the determinants of DM-TB comorbidity.

### Authors’ Contribution:

**NK**: Project concept & study design, literature review, manuscript write up and take the overall responsibility & accountable for the accuracy or integrity of the work.

**FA:** Research Supervisor, Formal analysis and final approval of the manuscript.

**FMQ:** Data collection, statistical analysis and interpretation of results, reviewed and edited the manuscript.
